# Mitochondrial gene signature in the prefrontal cortex for differential susceptibility to chronic stress

**DOI:** 10.1038/s41598-020-75326-9

**Published:** 2020-10-27

**Authors:** Meltem Weger, Daniel Alpern, Antoine Cherix, Sriparna Ghosal, Jocelyn Grosse, Julie Russeil, Rolf Gruetter, E. Ronald de Kloet, Bart Deplancke, Carmen Sandi

**Affiliations:** 1 Laboratory of Behavioral Genetics, Brain Mind Institute, École Polytechnique Fédérale de Lausanne, 1015 Lausanne, Switzerland; 2 Laboratory of Systems Biology and Genetics, Institute of Bioengineering, École Polytechnique Fédérale de Lausanne, 1015 Lausanne, Switzerland; 3 Laboratory for Functional and Metabolic Imaging, École Polytechnique Fédérale de Lausanne, 1015 Lausanne, Switzerland; 4 Departement of Endocrinology and Metabolic Disease, Leiden University Medical Center, Leiden, The Netherlands; 5 Institute for Molecular Bioscience, The University of Queensland, St. Lucia, QLD 4072 Australia; 6 Swiss Institute of Bioinformatics, 1015 Lausanne, Switzerland; 7 Present Address: Nuffield Department of Clinical Neurosciences, John Radcliffe Hospital, University of Oxford, Oxford, OX3 9DU England, UK

**Keywords:** Stress and resilience, Neuroscience

## Abstract

Mitochondrial dysfunction was highlighted as a crucial vulnerability factor for the development of depression. However, systemic studies assessing stress-induced changes in mitochondria-associated genes in brain regions relevant to depression symptomatology remain scarce. Here, we performed a genome-wide transcriptomic study to examine mitochondrial gene expression in the prefrontal cortex (PFC) and nucleus accumbens (NAc) of mice exposed to multimodal chronic restraint stress. We identified mitochondria-associated gene pathways as most prominently affected in the PFC and with lesser significance in the NAc. A more detailed mitochondrial gene expression analysis revealed that in particular mitochondrial DNA-encoded subunits of the oxidative phosphorylation complexes were altered in the PFC. The comparison of our data with a reanalyzed transcriptome data set of chronic variable stress mice and major depression disorder subjects showed that the changes in mitochondrial DNA-encoded genes are a feature generalizing to other chronic stress-protocols as well and might have translational relevance. Finally, we provide evidence for changes in mitochondrial outputs in the PFC following chronic stress that are indicative of mitochondrial dysfunction. Collectively, our work reinforces the idea that changes in mitochondrial gene expression are key players in the prefrontal adaptations observed in individuals with high behavioral susceptibility and resilience to chronic stress.

## Introduction

Chronic stress is a major risk factor for the development of psychopathologies, such as depression^1–3^, a severe mental disease with a high prevalence and limited effective treatment possibilities^4,5^. Despite extensive clinical and preclinical research efforts carried out to date (e.g.,^6–12^), there is still a lack of understanding about the specific biological changes that link chronic stress with depressive symptoms.

Brain regions, such as the prefrontal cortex (PFC) and the nucleus accumbens (NAc) that are implicated in depression pathogenesis^11,13–16^ show high susceptibility to display structural and functional alterations under exposure to chronic stress^12,17–19^. In the search for potential mechanisms underlying those changes, several studies have reported gene expression changes in the PFC and NAc in both, depressive patients^16,20–22^ and rodent models of chronic stress^23–25^. A very recent comparative study has reported considerable overlap between transcriptional signatures in the PFC and NAc in human major depressive disorder and in three mouse chronic stress models, validating the use of these animal approaches to model depression^26^. Interestingly, only the enrichment of genes involved in mitochondrial function in both PFC and NAc was commonly shared between human depression and every mouse model, whereas for all other shared gene pathways each mouse model captured distinct aspects of major depression abnormalities^26^. While these findings suggest that alterations in brain mitochondrial-related gene expression may be a critical evolutionarily conserved pathway linking chronic stress with depression, this study notably did not follow up on the mitochondrial data^26^.

Mitochondria are multifunctional life-sustaining organelles responsive to life stress and increasingly recognized as potential intersection points between psychosocial experiences and biological stress responses^27,28^. For example, stress-induced mitochondrial impairments leading to decreased ATP synthesis and/or excessive production of reactive oxygen species (ROS) may affect behavior by interfering with neuroplasticity and causing neurotoxicity^28–30^. Indeed, mitochondrial dysfunction is emerging as a vulnerability factor for human depression^31,32^ and stress-related psychopathologies^28,30^. However, information regarding the precise stress-induced changes in mitochondria-associated genes in brain regions relevant for depression symptomatology, and how they relate to depression-related behavioral manifestations, is scarce. Only a few studies have investigated alterations in the transcriptional expression of mitochondria-associated genes in the brain of major depression disorder (MDD) subjects^33^, including the PFC^34^. In rodent models of chronic stress, a recent multi-omics study including genome-wide analyses identified mitochondrial-related alterations in gene expression in the bed nucleus of stria terminalis, an anxiety-related brain region^35^.

Here, we performed a genome-wide transcriptomic study using bulk transcriptomics to assess and define mitochondrial gene expression signatures in the PFC and NAc upon chronic stress. We firstly investigated whether mitochondrial gene sets and pathways would show enriched differential expression in the PFC and NAc between mice submitted to multimodal chronic restraint stress (mCRS) and unstressed control (CTR) mice. As our results revealed prominent chronic stress-induced alterations in mitochondrial pathways, we then performed a detailed analysis to understand to which extent nuclear (nDNA) and mitochondrial DNA (mtDNA)-encoded mitochondria-related genes were affected in these brain regions. In addition, to assess whether the detected mitochondrial gene expression changes are a chronic stress feature that generalizes to other protocols, we reanalyzed a previously published gene expression data set of PFC and NAc from chronic variable stress (CVS) mice^20^. Moreover, to provide translational implications of our findings, we compared our data with differential gene expression in major depression disorder (MDD) subjects^20^. Lastly, we provide evidence that the here defined mitochondrial gene expression signature reflects the mice behavioral profiles and their susceptibility to chronic stress.

## Results

### The mCRS protocol leads to changes in physiology and behavior

**Figure 1:**
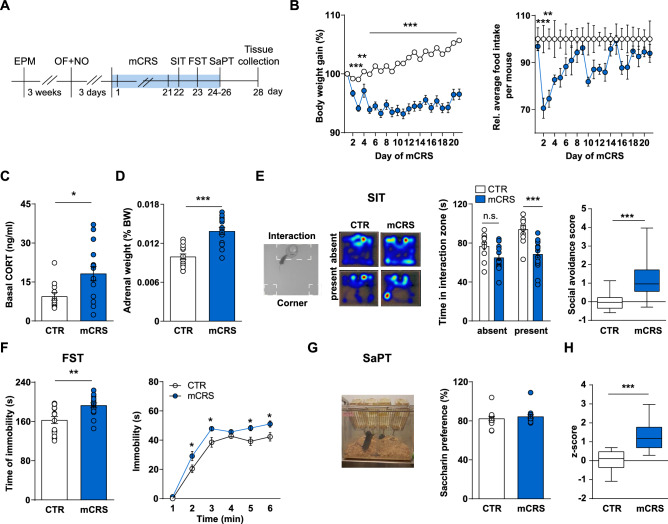
Effects of chronic stress in physiology and behavior. (**A**) Experimental timeline: Mice were randomized in two groups according to body weight and performance in the elevated plus maze (EPM) and open field and novel object (OF + NO) tests: unstressed controls (CTR) and mice submitted to multimodal chronic restrain stress (mCRS) N = 16/group. From mCRS day 21, they were tested in the social Interaction (SIT), forced swim (FST), and saccharine preference (SaPT) tests. Tissue was collected on day 28, 4 days after the last stress session. (**B**) Changes in body weight (stress effect: F_1,30_ = 94.67, *p* < 0.0001; interaction: F_20,600_ = 35.42, *p* < 0.0001; repeated measures two-way Anova, Sidak’s multiple comparison test; n = 16/group) and food intake weight (stress effect: F_1,6_ = 4.64, *p* = 0.0747; interaction: F_20,120_ = 3.841, *p* < 0.0001; repeated measures two-way Anova, Sidak’s multiple comparison test; n = 16/group). (**C**) Post-mCRS basal corticosterone (CORT) levels on day of sacrifice (Mann–Whitney U-test, two-tailed; n = 12 CTR, n = 15 mCRS). (**D**) Post-mCRS adrenal glands weight (Unpaired t-test, two-tailed; n = 15 CTR, n = 16 mCRS). (**E**) SIT: top-down view of the arena and representative heat maps when a social target is absent or present. Barplots indicate the time in the interaction zone (ST presence: F_1,25_ = 7.88, *p* = 0.0096; stress effect: F_1,25_ = 25.16, *p* < 0.0001; interaction: F_1,25_ = 3.352, *p* = 0.0791; repeated measures two-way Anova, Sidak’s multiple comparison test; n = 11 CTR, n = 16 mCRS) and the social avoidance score (Unpaired t-test, two-tailed; n = 11 CTR, n = 16 mCRS). (**F**) FST: Immobility time total (Unpaired t-test, two-tailed; n = 16/group) and per min (stress effect: F_1,30_ = 15.67, *p* = 0.0004; interaction: F_5,150_ = 1.553, *p* < 0.1769; repeated measures two-way Anova, Sidak’s multiple comparison test; n = 16/group). (**G**) SaPT: Saccharin preference over 3 days (Unpaired t-test, two-tailed; n = 16/group). (**H**) Integrated behavioral z-score (computed from z-scores of SIT, FST, and SaPT; Unpaired t-test, two-tailed; n = 11 CTR, n = 16 mCRS). Data are displayed as mean ± SEM. *, *p* < 0.05, **, *p* < 0.01, ***, *p* < 0.001.

Mice were randomized into CTR or mCRS groups following matching according to their body weight and anxiety-like behaviors (i.e., percent time in the open arms of the EPM; see experimental schedule in Fig. 1A). The OF and NO tests confirmed that there were no a priori group differences in anxiety, locomotion, and exploration (Fig. S1A,B). During exposure to mCRS, stressed mice showed reduced body weight gain and lower food intake at the beginning of the stress paradigm than CTRs (Fig. 1B). Stress also led to increased basal blood CORT levels, as measured four days after the end of the mCRS protocol (Fig. 1C) and increased adrenal glands’ weight (Fig. 1D).

**Figure 2:**
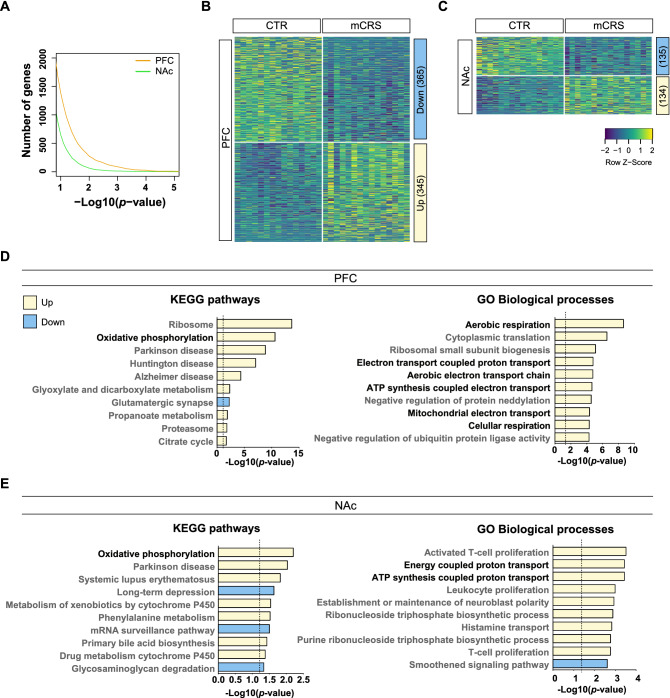
Transcriptional gene expression changes in the PFC and NAc of mice following mCRS. (**A**) Number of differentially expressed genes (CTR vs. mCRS) in the in the PFC (n = 15/group) and in the NAc (n = 16/group) from mCRS mice and unstressed CTRs in function of the *p* value. (**B**) Heatmap of normalized mRNA expression levels of genes in the PFC and (**C**) in the NAc. Yellow, high expression; blue, low expression. (**D**) Gene set enrichment analyses of differentially expressed genes in the PFC and (**E**) in the NAc. Mitochondrial pathways are highlighted in black font. Yellow bars are up-regulated pathways and blue bars are down-regulated pathways.

At the behavioral level, and as compared to CTRs, mice exposed to mCRS showed: (1) decreased sociability, as indicated by their reduced time spent in the social interaction zone and increased social avoidance score (Fig. 1E); (2) increased immobility in the FST (Fig. 1F); and (3) unaltered saccharin preference (Fig. 1G). Importantly, the computed integrated z-score indicated that mCRS exposed animals showed a significant alteration when the performance across the different tested behavioral domains was considered (Fig. 1H).

### mCRS severely affects the PFC transcriptome and leads to changes in mitochondrial pathways

Next, we performed a hypothesis-free transcriptomic analysis to explore the effects of mCRS on PFC and NAc gene expression (see Table S1 for a list of differentially expressed genes in the PFC and NAc, and Fig. S2A, B for mapping efficiency). The overall number of differentially expressed genes in the PFC following mCRS exposure was higher than the number of differentially expressed genes in the NAc (Fig. 2A–C). The top 20 differentially expressed genes contained genes previously implicated in stress-vulnerability and/or depression (for details, see Fig. S2C,D), indicating that mCRS leads to pathological changes in the PFC and NAc transcriptomes. We then performed a gene set enrichment analysis to identify the most affected pathways in the PFC and NAc (Table S2) upon mCRS. The top 10 enriched pathways in the PFC, interestingly, included gene pathways implicated in mitochondrial energy synthesis, such as “oxidative phosphorylation”, “aerobic electron transport chain”, and “mitochondrial electron transport” (Fig. 2D). In line with these findings, translation (e.g., “ribosome” and “cytoplasmic translation”) as a cellular energy-depending process was also affected in the PFC (Fig. 2D). Gene pathways implicated in mitochondrial energy synthesis were also detected in the NAc (e.g., “oxidative phosphorylation”, “energy coupled proton transport”; Fig. 2E), although with a lower significance than in the PFC. These observations highlight profound transcriptional imbalances in mitochondrial gene expression upon mCRS particularly in the PFC.

### Gene expression profiles of mitochondria-associated genes following mCRS

**Figure 3:**
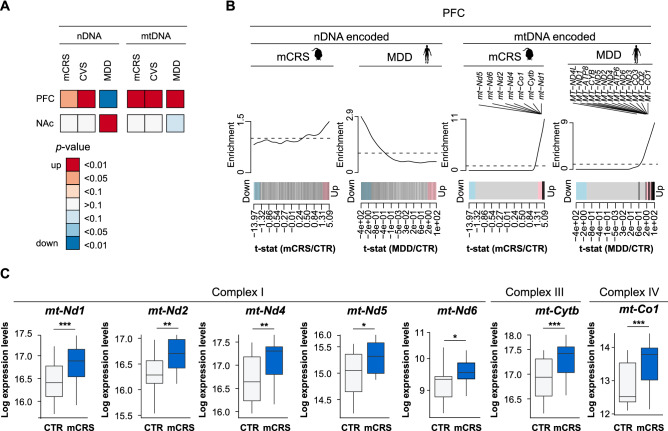
Mitochondria-related gene expression in chronically stressed mice and major depression subjects. (**A**) Enrichment analyses of mitochondria-associated genes encoded by nuclear DNA (nDNA) or mitochondrial DNA (mtDNA) in mCRS and unstressed CTR mice (n = 15/group, PFC; n = 16/group, NAc), chronic variable stress (CVS) mice (PFC: n = 19/group, NAc: n = 20/group) and *post-mortem* tissue of major depression disorder (MDD; n = 16, ventromedial (vm) PFC; n = 16, NAc) and healthy (CTR; n = 15, vmPFC; n = 17, NAc) subjects. Red, enrichment for up-regulation; blue, enrichment for down-regulation. Data of CVS mice and MDD subjects were reanalyzed from a previously published transcriptome dataset^20^. (**B**) Barcode plots for mitochondria-associated genes encoded by nDNA or mtDNA DNA in the PFC of mice exposed to mCRS and of MDD subjects. (**C**) Gene expression of mtDNA encoded genes in the PFC of mice (*p* values from generalized linear model). Data are displayed as mean ± SEM. *, *p* < 0.05, **, *p* < 0.01, ***, *p* < 0.001.

Our former analyses pointed to changes in mitochondrial pathways upon mCRS in the PFC, and to some extent in the NAc. We next focused on the mCRS-induced changes in mitochondria-associated genes to decipher the contribution of nDNA and mtDNA encoded genes. mCRS led to an up-regulation of mitochondria-associated genes encoded by both nDNA and mtDNA encoded genes in the PFC (Fig. 3A). A deregulation of mitochondria associated genes was not detected in the NAc (Fig. 3A). Specifically, all of the detected mtDNA encoded genes of the oxidative phosphorylation (OXPHOS) complexes I, III and IV (i.e., *mt-Nd1, mt-Nd2, mt-Nd4, mt-Nd5, mt-Nd6, mt-Cytb,* and *mt-Co1*) were up-regulated in the PFC of mCRS mice (Fig. 3B,C). Note that the other six mtDNA encoded genes of the OXPHOS complexes (i.e., *mt-ND3, mt-ND4L, mt-Co2, mt-Co3, mt-Atp6,* and *mt-Atp8*) were too low expressed to be reliably detected in our study*.* We verified the expression changes of selected mtDNA encoded genes by qRT-PCR and confirmed upregulation of the complex I genes *mt-Nd1* and *mt-Nd-3* (Fig. S3). To obtain information about the expression of complex V genes, we analyzed *mt-Atp6* expression and detected its up-regulation in the PFC of stressed mice as well (Fig. S3).

To evaluate whether the here detected mitochondrial gene expression changes are a chronic stress feature that generalizes to other chronic stress protocols, we also tested and showed that the up-regulation of both nDNA and mtDNA encoded genes is not solely specific to mCRS but can be also detected following CVS exposure (Fig. 3A, Fig. S4).

Furthermore, in a translational effort, we next tested whether the detected mitochondrial gene expression changes in chronically stressed mice are also present under conditions of MDD. For this end, we reanalyzed the data of *post-mortem* brain tissue from MDD patients and matched control subjects previously published by Labonte et al.^20^. For our comparison, we used the data from vmPFC (henceforth termed PFC) and NAc. Interestingly, there was a high overlap in mitochondria-associated gene expression between the data in mice and MDD subjects for mtDNA-encoded genes in the PFC (Fig. 3A,B). Therefore, altogether these analyses point to mtDNA encoded gene expression changes as particularly susceptible to be altered in the context of chronic stress and depression.

### Chronic stress leads to changes in mitochondrial function and metabolism

**Figure 4:**
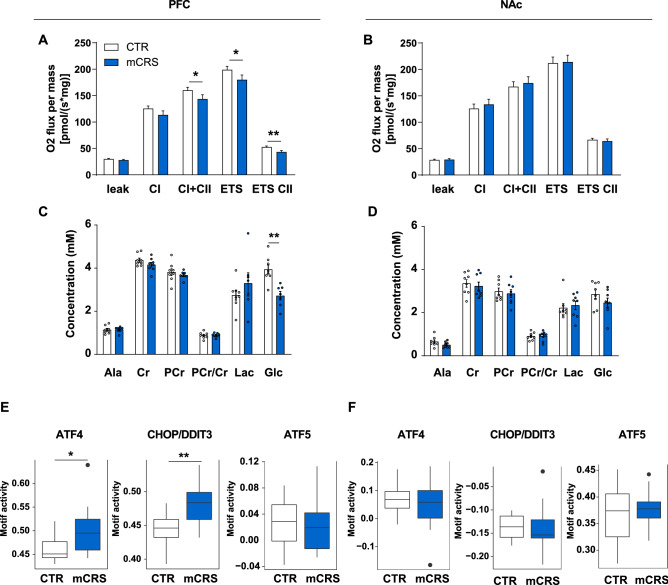
Effects of chronic stress on mitochondrial respiration and metabolites, and in silico transcription factor activity analysis in the PFC and NAc. (**A**) Ex vivo analysis of mitochondrial respiration in the PFC (*p* values from linear mixed models; n = 16 CTR, n = 15 mCRS). (**B**) Ex vivo analysis of mitochondrial respiration in the NAc (*p* values from linear mixed models; n = 16/group). (**C**) In vivo ^1^H-MRS measurements of energy metabolites in the PFC and (**D**) in the NAc (*p* values from linear models; n = 8/group). Abbreviations: Ala, alanine; Cr, creatine; PCr, phosphocreatine; Lac, lactate; Glc, glucose. (**E**) In silico analysis of BRB-seq data to determine potential transcription factor binding sites of ATF-5, ATF-4 and CHOP/DDIT3 in the promoters of differentially expressed genes in the PFC upon mCRS (Unpaired t-test, two-tailed; n = 15/group) and (**F**) in the NAc upon mCRS (Unpaired t-test, two-tailed; n = 16/group). Data are displayed as mean ± SEM. *, *p* < 0.05, **, *p* < 0.01.

The transcriptional changes in mitochondria associated genes indicated the presence of mitochondrial dysfunctions in mCRS mice. Thus, we assessed whether mCRS impacts mitochondrial function in the PFC and the NAc by addressing mitochondrial respiration capacity ex vivo. Our results showed a reduction in mitochondrial respiration capacity in mCRS mice in the PFC (Fig. 4A) but no changes in the NAc (Fig. 4B). To gain more insights on the impact of mCRS on brain metabolism, we performed ^1^H-magnetic resonance spectroscopy (^1^H-MRS; Fig. S5) and evaluated energy-related (i.e., creatine, phospho-creatine, glucose) metabolites, including glycolytic (i.e., alanine, lactate) ones^36^. Levels of glucose (Glc), the brain’s primary energy source, were decreased in the PFC of mCRS mice (Fig. 4C). No mCRS-induced changes in metabolites were detected in the NAc (Fig. 4D).

Next, we tested whether mCRS induces the mitochondrial unfolded stress-response (UPR^mt^) which is initiated by the transcription factors ATF-5, ATF-4 and CHOP/DDIT3^37–39^ upon mitochondrial dysfunctions to maintain homeostasis^40^. To predict in silico for potential transcription factor binding sites and thus activity of ATF-5, ATF-4 and CHOP/DDIT3 (“predicted motif activity”) in the promoters of differentially expressed genes in the PFC and NAc upon mCRS, we scanned ± 500 bp around the annotated transcription start site of a gene. Interestingly, our analysis revealed that the predicted ATF-4 and CHOP/DDIT3 motif activity is higher in the PFC of mCRS mice compared to CTR animals (Fig. 4E). In contrast, no increased predicted motif activity of any of the tested transcription factors was detected in the NAc of mCRS mice (Fig. 4F), indicating that the UPR^mt^ via the ATF4 pathway due to mitochondrial dysfunctions is active in the PFC of mice following mCRS.

### mtDNA encoded gene expression in the PFC correlates with mice behavioral profiles

**Figure 5:**
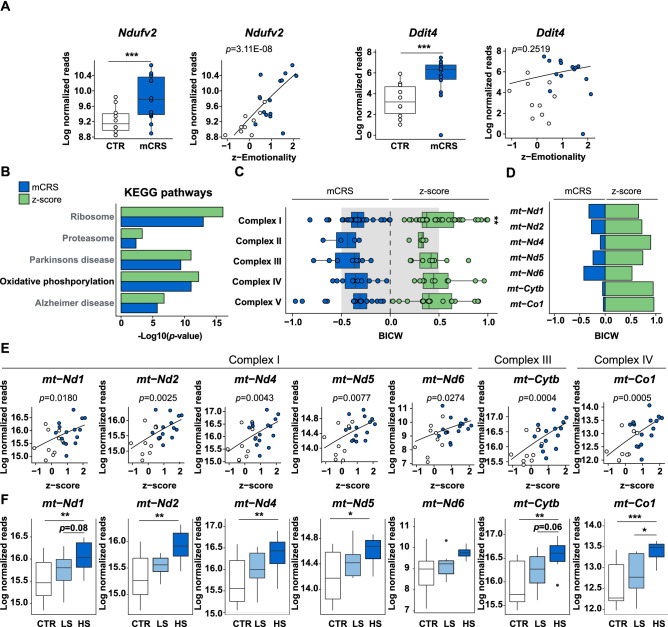
Model selection and stress susceptibility analysis of mCRS gene expression for CTR and high- and low-susceptible mCRS mice. (**A**) The gene expression profile of *Ndufv2* is better captured by a generalized linear model considering individual behavioral profiles (z-score) than a model that only considers the absence or presence of mCRS. On the other hand, *Ddit4* expression is better explained by the mCRS model than by the z-score model. (**B**) Gene pathways better described by the z-score than by mCRS. Oxidative phosphorylation is highlighted in black font. (**C**) Results of the model selection approach for genes associated with the indicated OXPHOS complexes. Genes associated with OXPHOS complex I better correlate with the integrated behavioral z-score than with mCRS. BICW, Bayesian information criterion weight. (**D**) Results of the model selection approach for detected mtDNA encoded genes. Gene expression of all detected mtDNA encoded genes are better explained by a model that relies on the integrated behavioral z-score rather than presence or absence of mCRS. (**E**) Correlational analyses between normalized mtDNA-encoded genes and integrated behavioral z-score. (**F**) Levels of mtDNA encoded genes from different OXPHOS complexes in CTR animals and mCRS mice grouped into high- (HS) or low-susceptibility (LS) to stress according to their integrated z-score (*p* values from generalized linear model). n = 11 CTR, n = 15 mCRS. Data are displayed as mean ± SEM. *, *p* < 0.05, **, *p* < 0.01, ***, *p* < 0.001.

To gather more information about whether the expression of mitochondria-associated genes in the PFC of mCRS mice can be related to mice behavioral profiles, we applied a model selection analysis of gene expression that considers the individual differences in stress and behavioral responses of the tested mice. Specifically, this method allows to extract genes either better correlating with 1) the treatment itself (i.e., mCRS) or with 2) the consequences of the treatment on behavior (i.e., z-score). In our analysis, the glucocorticoid-dependent mTOR regulator *Ddit4*^41^ would be an example for genes of group 1 and the mitochondrial gene *Ndufv2* would represent genes of group 2 (Fig. 5A, Table S1).

A gene set enrichment analysis of gene pathways better correlating with the behavioral profiles of mCRS mice led to the observation that “oxidative phosphorylation” is one of the pathways that is better explained by individual behavioral changes (z-score) than simply the absence or presence of mCRS (Fig. 5B, Table S2). A more detailed analysis of the OXPHOS complex genes showed that genes of complex I, but not of the other complexes, correlate better with the integrated behavioral z-score than with mCRS (Fig. 5C). In fact, in our study all detected mtDNA encoded genes correlated better with the z-score than with mCRS (Fig. 5D,E). In addition, we reevaluated our data by separating mCRS animals according to their susceptibility to stress [i.e., high- (HS) versus low-susceptible (LS), in accordance with^42^]. This approach showed that mCRS causes increased expression of mtDNA encoded genes only in HS but not in LS animals (Fig. 5F).

## Discussion

Mitochondrial dysfunction has been postulated as a key etiopathology factor for the development of chronic stress-induced psychopathological alterations, including depression^28,30^. Neural adaptations to chronic stress are energetically costly and mitochondria are ideally positioned to contribute to them by providing energy and stress signaling molecules^28,30^. However, systematic studies describing the precise alterations in mitochondria-associated genes in the brain following exposure to chronic stress remain scarce. In this study, using a genome-wide hypothesis-free transcriptomic approach, we identified mitochondria-associated gene pathways among the categories showing the most prominent responses in the PFC and to a lesser extent in the NAc following chronic stress exposure in mice. Most importantly, we revealed that mtDNA genes coding for different subunits of the OXPHOS complexes in the PFC as particularly affected by stress, and showed that their upregulation is particularly observed in stress-vulnerable mice.

First, we confirmed that our multimodal chronic restraint stress protocol leads to reduced sociability and increased passive coping responses under adversity, two behavioral alterations also typically observed in depressive individuals^43–45^. Then, the computation of the number of genome-wide stress-responsive genes, including mitochondria-associated ones, pointed at a higher reactivity of the PFC to stress than the NAc. This observation is in line with other reports showing that, at the transcriptional level, the PFC is more severely affected than the NAc by chronic stress or major depression^20,24,46^. Rodent studies have reported opposite effects of chronic stress in neuronal structure in these two brain regions, frequently indicating decreases in spine density and dendritic complexity in the PFC^47–50^ while increase in the NAc^49,51^ (but see^52^). However, a note of caution should be added here, not only because the evidence is mixed as indicated above, but also because different cell types are involved in the referred studies. While data from the NAc has been obtained from the analysis of GABAergic projection neurons (i.e., medium spiny neurons), the atrophy described in the PFC refers to excitatory pyramidal cells. In fact, opposite results (i.e., dendritic hypertrophy) have been found in the medial PFC when analyzing the effects of chronic stress in a subpopulation of interneurons^53^. However, the relatively lower susceptibility of the NAc to show transcriptomic changes following chronic stress, including mitochondria-associated ones, contrasts with the highest vulnerability shown by the NAc than the PFC to display changes in neurometabolic markers following stress^18^. A possible reason for this discrepancy may be the different stress protocols used (i.e., 10-days chronic social defeat^18^ vs. the here applied 3-week mCRS). Note, however, that a 3-way chronic restraint protocol results as well in major metabolic changes in the NAc in stress-vulnerable mice^54^. In the future, it will be important to integrate transcriptomic and metabolic analyses in these two brain regions, ideally at the cell-type level, to further our understanding of stress-induced metabolic regulation at the transcriptional level.

Mitochondria are multifunctional life-sustaining organelles that have as a major function to perform OXPHOS, the metabolic pathway in which the electron transport chain, through its complexes I–IV, exchanges protons and electrons to eventually produce adenosine triphosphate (ATP) by complex V. Subunits of the mitochondrial complexes of OXPHOS are encoded not only by nDNA but also by mtDNA^28^. Our mitochondria-focused analysis showed that mCRS mice exhibited an up-regulated expression of mtDNA encoded genes of virtually all OXPHOS complexes, except for complex II. We furthermore showed that mtDNA encoded genes are also up-regulated in the PFC of mice subjected to another chronic stress paradigm^20^. These results align well with proteomic data indicating chronic stress-induced changes in the levels of proteins involved in mitochondrial transport and OXPHOS in the PFC^55^. Likewise, chronic stress was also shown to lead to up-regulated expression of the mtDNA-encoded complex I gene mt-*ND6* in the rat hippocampus^56^. Altogether, these findings, strongly supported the notion that an upregulated expression of mtDNA encoded genes is a common response to chronic stress. Importantly, mouse gene deletion approaches involving genes of the mitochondrial OXPHOS complexes were shown to lead to alterations in physiological stress responses^27,57^.

Importantly, we found stress-induced increases in the expression of genes coding for subunits of different complexes of the OXPHOS machinery in the PFC along with decreased levels of the energy metabolite glucose and reduced maximal capacity of mitochondrial respiration as evaluated ex vivo. Diminished mitochondrial respiration in the PFC was also reported for other chronic stress models^58^ and in fibroblasts from major depressive disorder patients^59^. Glucose levels are also typically found decreased in the brain of depressed patients^60–62^ and following chronic stress in rodents^63^, in association with a reduction in glucose transporters expression^63^. Given that glucose is the main source of energy for the mammalian brain^64^, through a key contribution to OXPHOS processes^65^, the observed increases in genes coding for OXPHOS complexes subunits may be an allostatic response to compensate for reduced fuel^28,66,67^. However, given that our neurobiological endpoints were only measured after chronic stress exposure, we cannot discard that they are not a reaction to stress-induced enhancements in mitochondrial function potentially observed at earlier time points^56,68^, particularly in the PFC^69^. Eventually, the reported compensatory changes could represent responses to the “allostatic load” induced by chronic stress^70^. Such a view would reconcile the reductions in dendrite complexity and spine density typically observed following chronic stress in the PFC^47,48^ and the increased neuronal vulnerability triggered by elevated rate of mitochondrial OXPHOS^71,72^. Increased rate of mitochondrial OXPHOS would lead to enhanced production of ROS making neurons more vulnerable and less capable of sustaining complex axonal arborization^71,73^. This view is further reinforced by the fact that the increased expression of mtDNA encoded OXPHOS genes upon chronic stress relates with the behavioral profiles of the mice and was particularly observed in high-susceptible animals. Altogether, our data point to that mCRS leads to profound changes in mitochondrial outputs in the PFC that are indicative for mitochondrial dysfunction in this brain region.

Our findings of high transcriptional susceptibility of mitochondrial pathways to stress align well with the recent observation of similar marked dysregulations across three mouse models of chronic stress and in human MDD both in the PFC and in the NAc^26^. Here, we go beyond those observations and provide translational evidence for similar upregulation of mtDNA encoded OXPHOS genes in the PFC of subjects with major depression disorder, a new finding we made by reanalyzing a previously published transcriptome data set^20^. Altogether, these findings reinforce the idea that changes in mitochondrial gene expression are key players in the prefrontal adaptations observed in individuals with high behavioral susceptibility to chronic stress.

In the future, it will be important to perform similar mitochondrial analyses as the ones included in the current study to investigate chronic stress effects in other brain regions critically involved in the brain response to chronic stress and MDD, such as the orbitofrontal cortex (OFC), hippocampus and amygdala^74,75^. These studies will be particularly relevant to understand the link between mitochondria and stress-induced changes in neuronal structure as, in contrast to the hypotrophy induced by stress in the medial PFC^47–50^ or in the hippocampus^76,77^, the basolateral amygdala^78^ (but see^79^) and the OFC show dendritic hypotrophy^80^ following similar stress protocols. In addition, future functional studies will be needed to elucidate the underlying molecular mechanisms of how the herein defined mitochondrial gene expression changes can act as a vulnerability factor for the development and/or pathology of stress-induced depression. Some evidence from the literature points to glucocorticoids via the glucocorticoid receptor (GR) as potential mediators. Previous studies have shown that both mitochondrial function^81^ and glucocorticoid actions^82,83^ in the NAc is important for the establishment of social dominance in rats, a considered vulnerability factor for depression^84^. Moreover, the GR implicated in the mesocorticolimbic dopamine system with chronic stress vulnerability^85,86^, and showed that the GR has the capability to translocate into brain mitochondria^68,87,88^ and to bind on the mitochondrial genome^56,89^ in a glucocorticoid-dependent manner^56^. It will also be important to test whether mitochondrial-targeted interventions known to ameliorate stress and anxiety responses^54,90–92^ would reverse both the behavioral and mtDNA transcriptional changes observed in stress-vulnerable mice.

In summary, we here defined the mitochondrial gene signatures upon chronic stress in the PFC and NAc, two key brain regions implicated with stress-vulnerability and depression. Our data point to a higher susceptibility of the PFC to chronic stress on a transcriptional level than of the NAc. Moreover, our studies indicate that chronic stress induces a conserved response in mtDNA encoded gene expression in the PFC. Future studies will elucidate how these mitochondrial gene signatures might mediate differential susceptibility to chronic stress.

## Material and methods

### Animals

Experimental male 6-week old C57Bl6/J mice and retired old-breeder CD1 mice serving as social targets for the SIT test were purchased from Charles River Laboratories. Experimental animals were housed four per cage (except for the CD1 mice that were due to the experimental design under single-housed conditions) and were allowed to acclimate to the vivarium for at least one week. Animals were handled for at least 1 min per day for three subsequent days and then were weighted upon arrival and then weekly to ensure good health. Animals were kept under standard housing conditions on corn litter in a temperature- (23 ± 1 °C) and humidity- (40%) controlled animal room with a 12 h light/dark cycle (7 am–7 pm) with ad libitum access to food and water. All animal procedures were conducted in accordance with the regulations and approved by the Cantonal Ethics Committee of the Canton Vaud, Switzerland.

### Behavioral and physiological characterization

Mice (7 weeks old) were randomized in two equivalent groups according to their body weight and performance in the elevated plus maze (EPM). They were then exposed at the age of 10 weeks to open field and novel object (OF + NO) tests to verify the effectiveness of the randomization. Starting at an age of 11 weeks one group of mice was then exposed to chronic multimodal chronic restraint stress (mCRS; n = 16), the other was kept as unstressed controls (CTR; n = 16). Body weight and food intake was measured daily before stress exposure. Mice (14 weeks old) were then tested in the social interaction test (SIT), the forced swim test (FST), and the saccharin preference test (SaPT). Trunk blood was collected during sacrifice (15 weeks old) for basal plasma corticosterone (CORT) measurements and the adrenal glands were dissected to determine adrenal glands’ weight.

### EPM

The EPM was performed under baseline conditions to randomize animals into experimental groups according to their anxiety levels, a vulnerability factor for depression^93^. The apparatus consisted of black PVC with a white floor with two open arms and two closed arms (30 × 5 cm) placed 65 cm above the floor. Light conditions during the testing were 12 lx in the open arms and 3–4 lx in the closed arms. Experimental animals were placed into the center of the EPM and exploratory behavior were analyzed for 5 min with a video-tracking software (Ethovision 11.0 XT, Noldus, Information Technology). The time an experimental animal spent in the open arms, center, and closed arms was measured.

### OF + NO tests

The OF + NO tests were performed under light conditions of 7 lx. For the OF test, mice were placed in a white open field arena (50 × 50 cm) and were allowed to explore the arena for 10 min. For the NO test, experimental animals were then provided an empty water bottle as a novel object into the center of the arena, and were monitored for an additional time of 5 min. Via a video tracking system (Ethovision 11.0 XT, Noldus, Information Technology) the times an animal spend at the different zones of the arena (wall, intermediate, center) and the total distance travelled was calculated.

### mCRS paradigm

The mCRS paradigm includes daily restraint of mice in 50 ml falcon tubes equipped with air holes under changing conditions. The order was as follows: (1) 2 h restraint (9 to 11 am) at 89 lx light; (2) 3 h restraint (10 am to 1 pm) in darkness; (3) 1 h restraint (3 to 4 pm) at 89 lx light and loud music (65–70 dB); (4) 2 h restraint (9 to 11 am) at 144 lx light; (5) 1 h restraint (4 to 5 pm) at 89 lx light and slight jostling on a shaker; (6) 2 h restraint (10 am to 12 pm) under stroboscope light; (7) 40 min restraint (5 to 5:40 pm) at 89 lx light in the presence of 2,3,5-Trimethyl-3-thiazoline (TMT) odor; 8) 2 h restraint (9 to 11 am) in darkness; 9) 2 h restraint (9 to 11 am) at 144 lx light and loud music (65–70 dB); (10) 3 h restraint (10 am to 1 pm) at 89 lx light; (11) 3 h restraint (1 to 3 pm) at 144 lx light; (12) 2 h restraint (9 to 11 am) at stroboscope light; (13) 30 min restraint (10 to 10:30 am) at 89 lx light in the presence of TMT; (14) 1 h restraint (4 to 5 pm) at 89 lx light and slight jostling on a shaker; (15) 3 h (10 am to 1 pm) at 89 lx light; (16) 2 h (2 to 4 pm) at 144 lx light; (17) 2 h restraint (9 to 11 am) at 89 lx light and loud music (65–70 dB); (18) 1 h restraint (3 to 4 pm) under stroboscope light; (19) 40 min restraint (1 to 1:40 pm) at 144 lx light in the TMT odor; (20) 1 h restraint (10 to 11 am) at 89 lx light and slight jostling on a shaker; (21) 2 h restraint (9 to 11 am) at 89 lx light. After 21 days, 30 min of restraint stress at 89 lx light was continued in the afternoon between the behavioral tests to avoid recovery of the animals from the stressor.

### SIT

The SIT^94^ consisting of two consecutive sessions of 2.5 min was performed in a white open field arena (42 × 42 cm) at red light conditions. During the first session, animals were allowed to explore the arena including an empty wire mesh. During the second session, a CD1 mouse separated with a wire mesh was introduced into the arena as a social target. With a video tracking system (Ethovision 11.0 XT, Noldus, Information Technology) the time an experimental animal spent in the interaction zone (24 × 14 cm) and in the corner zones (each 9 × 9 cm) was measured. Four wild-type animals had to be removed from the analysis due to technical reasons such as an early stopping of the video recording. The social avoidance score was calculated with the following parameters as described in^95^: (1) the time spend in the interaction zone in the presence of the social target, (2) the time spend in the corner zones in the presence of the social target, (3) the social interaction ratio (time spent in the interaction zone in the presence of the social target/time spent in the interaction zone in the absence of the social target), (4) the corner zone ratio (time spent in the corner zones in the presence of the social target/time spent in the corner zones in the absence of the social target).

### FST

The FST^96^ was performed for 6 min under light conditions of 90 lx. Mice were placed in 5 L beakers with 3.5 L of tap water at a temperature of 23–25 °C. Immobility time (the absence of any movement except to keep the animal head above the water) during the last 4 min of the session was scored manually.

### SaPT

For the SaPT, animals were transferred into new home cages with two drinking bottles containing drinking water to habituate them to the new set-up. The next day, one of the bottles was replaced with a 0.05% saccharin (Sigma, #S1002) solution and the weight of the water bottles was measured every day for three subsequent days. The bottles were interchanged after each weight measurement to avoid the development of side preference of the animals. Total saccharin consumption was calculated as a percentage of amount of saccharin solution consumed divided by the total volume consumed.

### Behavioral z-score index

In order to have an integrated measure of behavioral phenotypes, we performed an integrated behavioral z-score composed of the integrated computation of respective z-normalization across behavioral tests. This approach allows standardizing observations obtained in different experiments, enhancing the reliability of the behavioral phenotyping and increasing analytical possibilities^97^. Specifically, our z-score was calculated by averaging the z-normalized raw data [(x-mean of CTR group)/STD of CTR group] obtained per mouse in the SIT (social avoidance), FST (time of immobility), and SaPT (% preference) tests. The z-score was determined for mice for which the behavioral parameters could be collected for all tests (i.e., 11 CTR, 16 mCRS mice). In agreement with previous studies^42^, we subdivided chronically stressed mice into low-susceptible (LS) and high-susceptible (HS) clusters according to their z-score. Thus, mCRS mice which fell into the standard deviation from the mean of the control group were designated as LS, as they exhibit comparable behavior to unstressed CTRs, and animals that fell outside were designated as HS.

### CORT levels

Trunk blood was collected (sufficient amounts were obtained for 12 CTR and 15 mCRS mice) in heparin-coated capillary tubes (Sarsted, Switzerland) and was centrifuged at 9400 × *g* at 4 °C for 4 min to obtain plasma. Basal plasma CORT levels were diluted 20 times and then were measured with an ELISA (Enzo Life Sciences, ADI-901-097 for corticosterone) according to the manufacturer’s protocol. Concentration values of CORT were calculated using a 4-parameter logistic fit.

### Adrenal glands weight

Adrenal glands were dissected (due to loss during tissue collection, not available for one CTR mouse) and measured to determine adrenal gland weights normalized with body weights of each tested animal [weight adrenal gland in g/body weight in g) * 100].

### Mitochondrial respiratory capacity

PFC and NAc from one hemisphere per animal, counterbalanced across samples [n = 16 CTR, n = 15 mCRS (due to loss during tissue collection, not available for one stressed animal) for PFC and n = 16/group for NAc], was used to measure mitochondrial respiration using the Oroboros Oxygraph 2 K (Oroboros Instruments, Innsbruck, Austria), as previously described^81^. Briefly, tissue was weight and homogenized in ice-cold respirometry medium (0.5 mM EGTA, 3 mM MgCl2, 60 mM potassium lactobionate, 20 mM taurine, 10 mM KH2PO4, 20 mM HEPES, 110 mM sucrose and 0.1% (w/v) BSA, pH = 7.1) with an eppendorf pestle. 2 mg of tissue was used to run a multisubstrate protocol at 37 °C to sequentially assess the various components of mitochondrial respiratory capacity through oxidative phosphorylation (OXPHOS): respiration due to complex I activity (Complex I) was measured by adding 5 mM ADP to a mixture of 2 mM malate, 10 mM pyruvate and 20 mM glutamate, followed by the addition of 10 mM succinate to subsequently stimulate complex II (Complex I + II). Respiration then was uncoupled to examine maximal capacitiy of the electron transport system (ETS) using the protonophore, carbonylcyanide 4 (trifluoromethoxy) phenylhydrazone (FCCP; successive titrations of 0.2 µM until maximal respiration rates were reached). The consumption in the uncoupled state due to complex II activity was determined by inhibiting complex I with the addition of 0.1 µM rotenone (ETS CII). In the last step, electron transport though complex III was inhibited to obtain the level of residual oxygen consumption (ROX) due to oxidating side reactions outside of mitochondrial respiration by adding 2 µM antimycin. The O_2_ flux obtained in each step of the protocol was normalized by the wet weight of the tissue sample used for the analysis and corrected for ROX.

### ^1^H-magnetic resonance spectroscopy (^1^H-MRS)

Another batch of mice (n = 8/group) was devoted to in vivo ^1^H-MRS measurements of brain metabolites, following procedures previously described in^18,54^. Briefly, all experiments were performed on a horizontal 14.1 T/26 cm bore animal MR scanner (Magnex Scientific, Abingdon, UK) using a homemade quadrature ^1^H-coil. The volume of interest (VOI) for spectroscopy was placed in the medial PFC (voxel size: 1.4 × 1.7 × 1.2 mm^3^) or in the bilateral NAc (voxel size: 4.1 × 1.4 × 1 mm^3^) after acquisition of a set of anatomical T_2_-weighted images for localization. Field homogeneity was adjusted using FAST(EST)MAP to reach a typical water linewidth of 15 Hz in medial PFC and 20 Hz in NAc^98^. Spectra were acquired with 20 blocks of 16 averages for medial PFC and 25 blocks of 16 averages for the NAc, leading to a scan duration of around 20 and 25 min respectively. After post-processing of the spectra, metabolite concentrations as well as the Cramér-Rao lower bounds (CRLB) were determined with LCModel using water as internal reference^99^.

### RNA isolation, cDNA synthesis, and quantitative RT-PCR

PFC and NAc from one hemisphere per animal, counterbalanced across samples, were snap frozen in liquid nitrogen until use for RNA extraction (n = 16/group). To this end, we used RNAqueous-Micro Total RNA Isolation Kit (ThermoFisher Scientific; #AM1931) and cDNA synthesis was done with the qScript cDNA SuperMix (QuantaBio; #733-1176). Quantitative RT-PCR (qRT-PCR) was performed using the SYBR Green PCR Master Mix (Applied Biosystems, Life Technologies, USA) at an ABI Prism 7900 Sequence Detection system (Applied Biosystems, Singapore) with eukaryotic elongation factor 1 (*Ef1a*) as a reference gene. Primer (Microsynth AG) sequences were as follows**:** Ef1a fw: tccacttggtcgctttgct, rv: cttcttgtccacagctttgatga; ND-1: fw: ggatgagcctcaaactccaa, rv: ggtcaggctggcagaagtaa; ND-3: fw: gcattctgactcccccaaat, rv: tgaattgctcatggtagtgga; ATP-6: fw: ccttccacaaggaactccaa, rv: ggtagctgttggtgggctaa. Gapdh: fw: tcaccaccatggagaaggc, rv: gctaagcagttggtggtgca.

### Bulk RNA-sequencing

The extracted RNA was used for performing the transcriptomic analysis from PFC and NAc of mCRS and unstressed CTR mice [n = 15/group for PFC (two samples had to be removed from the analysis due to poor sequencing quality); n = 16/group for NAc] using bulk RNA (BRB)-sequencing^100^. Briefly, each RNA sample was reverse transcribed in a 96-well plate using SuperScript II Reverse Transcriptase (Lifetech 18064014) with individual barcoded oligo-dT primers (Microsynth). Next, all the samples were pooled together, purified using the DNA Clean and Concentrator kit (Zymo Research #D4014), and treated with exonuclease I (NEB or New England BioLabs #M0293S). Double-stranded cDNA was generated by the second stand synthesis via the nick translation method. For that, a mix containing 2 μL of RNAse H (NEB, #M0297S), 1 μL of Escherichia coli DNA ligase (NEB, #M0205 L), 5 μL of E. coli DNA Polymerase (NEB, #M0209 L), 1 μL of dNTP (0 0.2 mM), 10 μL of 5 × Second Stand Buffer (100 mM Tris–HCl (pH 6.9, AppliChem, #A3452); 25 mM MgCl2 (Sigma, #M2670); 450 mM KCl (AppliChem, #A2939); 0.8 mM β-NAD Sigma, N1511); 60 mM (NH4)2SO4 (Fisher Scientific Acros, #AC20587); and 11 μL of water was added to 20 μL of ExoI-treated first-strand reaction on ice. The reaction was incubated at 16 °C for 2.5 h. Full-length double-stranded cDNA was purified with 30 μL (0.6 ×) of AMPure XP magnetic beads (Beckman Coulter, #A63881) and eluted in 20 μL of water. The Illumina compatible libraries were prepared by tagmentation of 5 ng of full-length double-stranded cDNA with 1 µL (11 μM) of in-house produced Tn5 enzyme. The final library was amplified 15 cycles and the fragments ranging 200–1000 bp were size-selected using AMPure beads (Beckman Coulter, #A63881) (first round 0.5 × beads, second 0.7 ×). The libraries were profiled with High Sensitivity NGS Fragment Analysis Kit (Advanced Analytical, #DNF-474) and measured with Qubit dsDNA HS Assay Kit (Invitrogen, #Q32851) prior to pooling and sequencing using the Illumina NextSeq 500 platform using a custom primer and the High Output v2 kit (75 cycles) (Illumina, #FC-404-2005). The library loading concentration was 2.2 pM and sequencing configuration as following: R1 6c/index 8 c/R2 70c.

### Transcriptomic analysis

To assess whether changes in mitochondrial gene expression are a common response to chronic stress, we made use of a publicly available transcriptome data set [GEO accession number: GSE102556;^20^]. To this end, we reanalyzed the raw reads of the mouse subseries of this study with a focus on mitochondrial associated gene expression by assessing differential gene expression in PFC (n = 19/group) and NAc (n = 20/group) between chronic variable stress and unstressed mice. To determine whether MDD subjects exhibit similar changes in mitochondrial gene signatures, we made use of a similar analysis pipeline and applied it on the subseries for MDD subjects of the aforementioned data set [GEO accession number: GSE102556;^20^]. We assessed differential gene expression between MDD [NAc: n = 16, 8 males and 8 females; ventromedial (vm) PFC: n = 16, 7 males and 9 females] and healthy subjects [NAc: n = 17, 9 males and 8 females; vmPFC: n = 15, 9 males and 6 females] with available medication status.

Reads produced in this study or publicly available raw reads were mapped with STAR 2.4.0 g^101^ onto GRCm38/mm10 and GRCh38/hg38 genome assembly for mouse and human data, respectively. Uniquely mapped reads were counted for each gene locus using htseq 0.6.1^102^. We normalized count data by size factor and applied a variance stabilizing transformation for visualization purposes as suggested by Anders et al.^103^. We utilized generalized linear model to assess differential gene expression with the help of the *DESeq2* package^104^. If not otherwise stated, we contrasted two groups for the mouse data [mCRS vs. CTR (data of this study), and CVS vs. CTR^20^] using a Wald test. To statistically assess differential gene expression between human MDD subjects and healthy controls we used a likelihood-ratio to correct differential gene expression for effects from age, RNA quality (RIN), alcohol abuse, medication status and gender as suggested^20^.

### Transcription factor activity analysis

To predict transcription factor binding sites of transcription factors in promoters of differentially expressed genes, we employed MotEvo 1.03^105^ and scanned ± 500 bp around the annotated transcription start site (TSS) of a gene. TSS definition were retrieved from^106^ and weight matrices were retrieved from Swiss regulon^107^. To compute transcription factor activity, we applied a penalized regression model described in Balwierz et al.^106^.

### Functional and gene set enrichment analysis

For gene set enrichment analysis we employed the *camera* function of the *limma* package^108^ on a pre-ranked gene lists defined from the differential gene expression analysis. Gene sets were derived from several sources including AmiGO^109^ for Gene Ontology Biological processes and KEGG^110^. The gene set of mitochondrial associated genes was retrieved from MitoCarta2.0^111^.

### Model selection

To assess whether a gene is better explained by the individual behavioral changes of an animal or the absence or presence of mCRS, we applied a model selection approach under the generalized linear model framework of *DESeq2*. To this end, we defined three models: (1) gene expression explained by z-score, (2) by presence or absence of mCRS or (3) no difference between animals. Each model was solved with generalized linear regression and Bayesian information criterion (BIC) was computed using the deviance of the fit to control for model complexity^112^. We calculated the Schwarz weight to assess model confidence for each model. This analysis was done with animals for which the z-score could be determined and for which transcriptome data were available (n = 10 CTRs, n = 15 mCRS).

### Statistics

Statistical analyses were performed with Prism 6.0 (Graphpad Software Inc.) and R (R Core Team, 2014). Unless otherwise stated, statistical significance was determined for the comparison of two groups by Student’s t-test for data following normal distribution or Mann–Whitney U-test for data following non-normal distribution. For analyses involving more than two groups, a 2-way analysis of variance (ANOVA) followed by Sidak’s post hoc test was applied. We log-transformed metabolite levels for spectroscopy and used a linear model to test for the contrast mCRS versus CTR. *p* values were corrected for multiple testing using the Benjamini–Hochberg method^113^. To test for differences between mCRS and CTR treated mice in respiration experiments, we constructed a linear mixed effects models using the R package *lme4*^114^. As the measurements for the mitochondrial respiration capacity experiments were performed in blocks of different days, we extracted the *p* values for the fixed effect treatment and controlled for day of measurement as a random effect. We reported the estimated marginal means of the model. All values represent the mean ± SEM. Asterisks indicate *p* value: *p < 0.05, **p < 0.01, ***p < 0.001.

## Supplementary information

Supplementary Information.

Supplementary Table 1.

Supplementary Table 2.

## Data Availability

The generated datasets of this study were made publicly available at the GEO repository (GEO accession number: GSE148629) and can be accessed via the following link: https://www.ncbi.nlm.nih.gov/geo/query/acc.cgi?acc=GSE148629.
