# Supplementary material for: Mitochondrial gene signature in the prefrontal cortex for differential susceptibility to chronic stress
Source: Sci Rep. 2020 Oct 27;10:18308. doi: 10.1038/s41598-020-75326-9 (PMC7591539; doi:10.1038/s41598-020-75326-9)
Supplement: Supplementary file 1 — Supplementary Information. [file 41598_2020_75326_MOESM1_ESM.pdf]

## Supplementary Information

# Mitochondrial gene signature in the prefrontal cortex for differential susceptibility to chronic stress

Meltem Weger[1,5], Daniel Alpern[2,6], Antoine Cherix[3,$], Sriparna Ghosal[1], Jocelyn Grosse[1], Julie Russeil[2], Rolf Gruetter[3], E. Ronald de Kloet[4], Bart Deplancke[2,6], and Carmen Sandi[1*]

[1]Laboratory of Behavioral Genetics, Brain Mind Institute, École Polytechnique Fédérale de Lausanne, 1015 Lausanne, Switzerland.

[2]Laboratory of Systems Biology and Genetics, Institute of Bioengineering, École Polytechnique Fédérale de Lausanne, 1015 Lausanne, Switzerland.

[3]Laboratory for Functional and Metabolic Imaging, École Polytechnique Fédérale de Lausanne, 1015 Lausanne, Switzerland.

[4]Departement of Endocrinology and Metabolic Disease, Leiden University Medical Center, Leiden, Netherlands.

[5]Institute for Molecular Bioscience, The University of Queensland, St. Lucia, QLD, 4072, Australia.

[6]Swiss Institute of Bioinformatics, 1015 Lausanne, Switzerland.

[*]Corresponding author: carmen.sandi@epfl.ch

[$] Current address: Nuffield Department of Clinical Neurosciences, John Radcliffe Hospital, University of Oxford, Oxford OX3 9DU, England.

[*]Corresponding author

Contact: carmen.sandi@epfl.ch

## Supplementary Figures

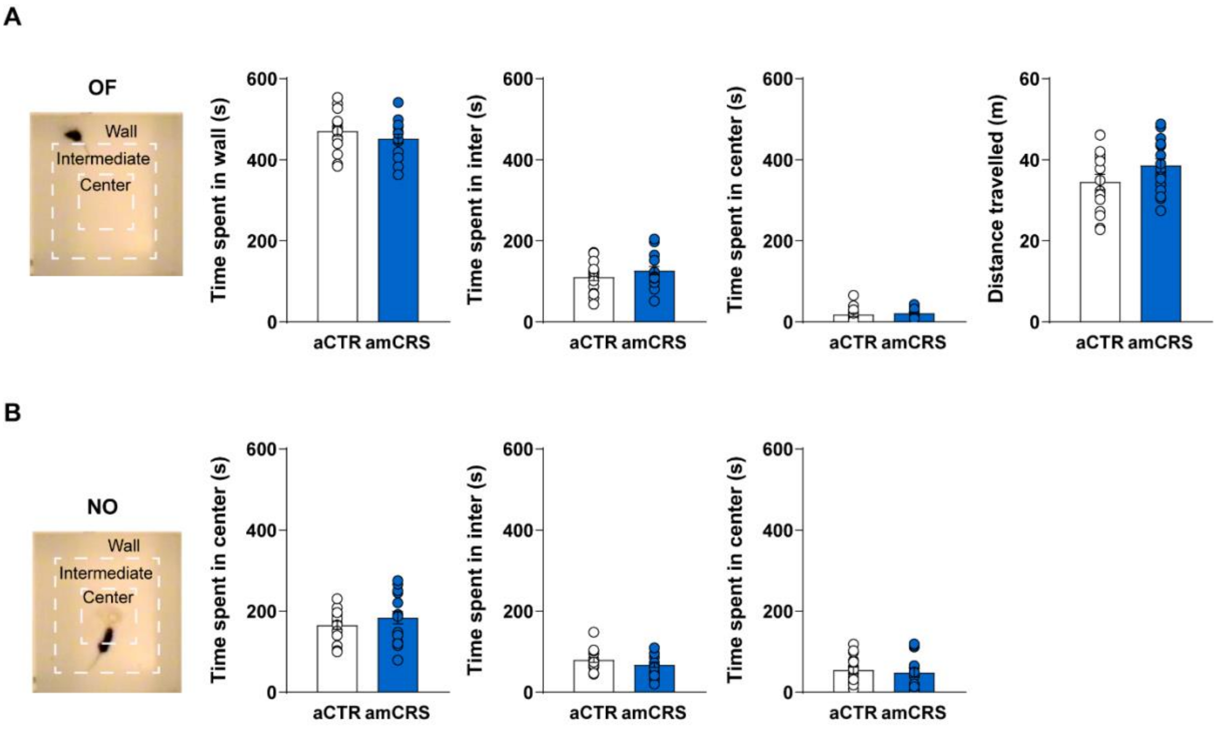


**Figure S1. Anxiety, locomotion and exploration levels in the two experimental groups under basal conditions.**

(**A**) Top-down view of the Open field (OF) test, delineated with zones. Experimental groups were separated into animals assigned to undergo mCRS (amCRS) or kept as unstressed control animals (aCTR) and were tested in the OF for their time spent in the center, intermediate (inter), and wall zones and for the distance travelled. **(B)** Top of the novel object (NO) test, delineated with zones. Animals were tested in the NO for their time spent in the center, intermediate (inter), and wall zones in the presence of an empty bottle as a novel object. Unpaired T-test, two-tailed; n=16/group. Data are displayed as mean ± SEM.

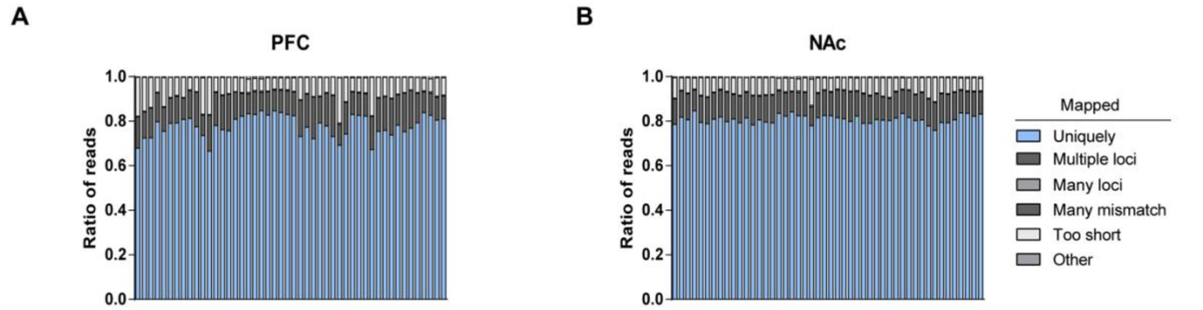


C

| PFC | | | | | |
|---|---|---|---|---|---|
| Accession number | Gene name | log2FoldChange | lfcSE | pvalue | padj |
| ENSMUSG00000107227 | Gm42559 | -23.16695465 | 1.639947757 | 2.60E-45 | 3.73E-41 |
| ENSMUSG00000085436 | Zfp335os | -20.87354746 | 1.758041897 | 1.63E-32 | 1.17E-28 |
| ENSMUSG00000045471 | Hcrt | -18.98901549 | 2.229854949 | 1.65E-17 | 7.90E-14 |
| ENSMUSG00000036357 | Gpr101 | 5.002824248 | 0.981915003 | 3.49E-07 | 0.001248888 |
| ENSMUSG00000028889 | Yrdc | -2.837714553 | 0.586426722 | 1.30E-06 | 0.003115009 |
| ENSMUSG00000049775 | Tmsb4x | 0.565838746 | 0.116725236 | 1.25E-06 | 0.003115009 |
| ENSMUSG00000108808 | Gm45204 | -6.259699975 | 1.340143308 | 3.00E-06 | 0.006135056 |
| ENSMUSG00000031790 | Mmp15 | -4.226969443 | 0.928199657 | 5.26E-06 | 0.009425546 |
| ENSMUSG00000097131 | D230017M19Rik | -5.901753271 | 1.310233649 | 6.66E-06 | 0.010224993 |
| ENSMUSG00000107643 | Gm43885 | 3.609030939 | 0.80388424 | 7.14E-06 | 0.010224993 |
| ENSMUSG00000113810 | Gm48259 | -6.54415445 | 1.472448024 | 8.81E-06 | 0.011475267 |
| ENSMUSG00000040373 | Cacng5 | 5.417597342 | 1.224322099 | 9.65E-06 | 0.011511604 |
| ENSMUSG00000074873 | AI606181 | -5.475041057 | 1.286373922 | 2.08E-05 | 0.022909449 |
| ENSMUSG00000038059 | Smim3 | 5.982568576 | 1.415439412 | 2.37E-05 | 0.024265048 |
| ENSMUSG00000063558 | Aox1 | -6.769953581 | 1.618985163 | 2.89E-05 | 0.027637715 |
| ENSMUSG00000066440 | Zfyve26 | 3.689494265 | 0.887171123 | 3.20E-05 | 0.028647501 |
| ENSMUSG00000030835 | Nomo1 | -0.964929783 | 0.236687291 | 4.57E-05 | 0.036327927 |
| ENSMUSG00000047507 | Baiap3 | -2.630474244 | 0.644181417 | 4.44E-05 | 0.036327927 |
| ENSMUSG00000020108 | Ddit4 | 2.046852959 | 0.506151901 | 5.26E-05 | 0.037636885 |
| ENSMUSG00000032511 | Scn5a | 5.350882072 | 1.319686292 | 5.02E-05 | 0.037636885 |

D

| NAc | | | | | |
|---|---|---|---|---|---|
| Accession number | Gene name | log2FoldChange | lfcSE | pvalue | padj |
| ENSMUSG00000111765 | Gm10635 | -19.14700128 | 2.032821371 | 4.56E-21 | 9.45E-17 |
| ENSMUSG00000051920 | Rspo2 | -6.98482278 | 1.088924017 | 1.41E-10 | 9.77E-07 |
| ENSMUSG00000052616 | Terb1 | -18.03298189 | 2.798400909 | 1.16E-10 | 9.77E-07 |
| ENSMUSG00000094707 | A830019P07Rik | 18.62023189 | 3.110110007 | 2.14E-09 | 1.11E-05 |
| ENSMUSG00000048416 | Mlf1 | 16.35890216 | 3.174011962 | 2.55E-07 | 0.001057356 |
| ENSMUSG00000032532 | Cck | 1.080100698 | 0.25234253 | 1.87E-05 | 0.064501396 |
| ENSMUSG00000105958 | Gm43319 | -5.326309052 | 1.254860612 | 2.19E-05 | 0.064882767 |
| ENSMUSG00000022206 | Npr3 | -5.806586102 | 1.388453823 | 2.89E-05 | 0.074876641 |
| ENSMUSG00000031283 | Chrdl1 | -3.791358077 | 0.974778036 | 0.000100468 | 0.231468075 |
| ENSMUSG00000048138 | Dmrt2 | -13.240958 | 3.510735453 | 0.000162229 | 0.336380891 |
| ENSMUSG00000097312 | Gm26870 | -2.820946186 | 0.755884769 | 0.000189981 | 0.35811464 |
| ENSMUSG00000106416 | Gm5857 | -4.158169197 | 1.144708346 | 0.000280673 | 0.484979979 |
| ENSMUSG00000030792 | Dkkl1 | 3.333752941 | 0.924611002 | 0.000311464 | 0.496785559 |
| ENSMUSG00000046230 | Vps13a | -1.405143346 | 0.395585615 | 0.000382229 | 0.519737323 |
| ENSMUSG00000051486 | Pcdhb11 | 7.124565639 | 2.012935409 | 0.000401051 | 0.519737323 |
| ENSMUSG00000071267 | Zfp942 | 2.55236944 | 0.719222833 | 0.000387008 | 0.519737323 |
| ENSMUSG00000029151 | Slc30a3 | 2.097134841 | 0.600075298 | 0.000474442 | 0.578679363 |
| ENSMUSG00000022309 | Angpt1 | 5.204498001 | 1.541764691 | 0.000736346 | 0.619449497 |
| ENSMUSG00000022364 | Tbc1d31 | -5.267753706 | 1.551201517 | 0.000683988 | 0.619449497 |
| ENSMUSG00000028691 | Prdx1 | 0.817863274 | 0.238640459 | 0.00060989 | 0.619449497 |

**Figure S2. Mapping efficiency of transcriptome study and top 20 differentially expressed genes in mCRS mice.**

(**A**) Bar diagram summarizing mapping statistics of the indicated samples for the PFC. **(B)** Bar diagram summarizing mapping statistics of the indicated samples for the NAc. **(C)** List with the top 20 differentially expressed genes in the PFC. **(D)** List with the top 20 differentially expressed genes in the NAc. Genes affected by mCRS can be implicated with stress-vulnerability and/or depression, including the mTOR pathway gene *Ddit4*/*Redd-1* [1], the gene of the hypocretin/orexin system *Hcrt* [2,3], the transcription factor *Mlf1* [4], and *Rspo2,* positive modulator of the wnt-signaling [5–8].

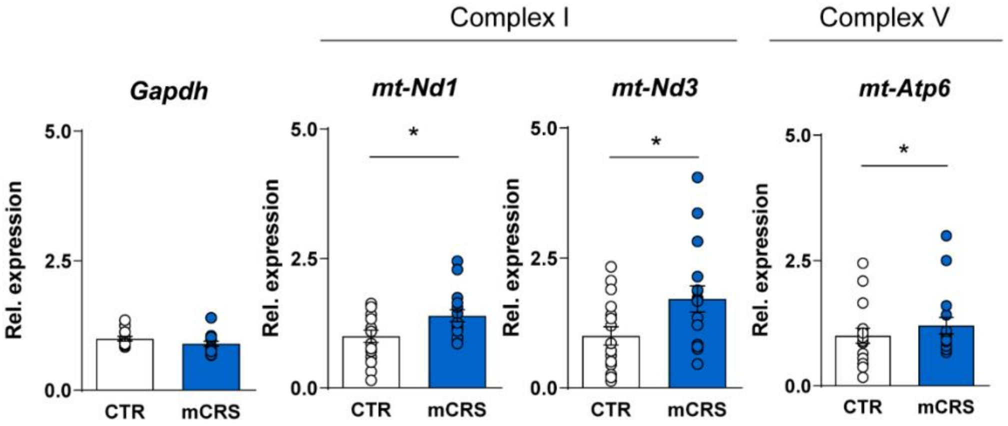


**Figure S3. qRT-PCR analysis of mitochondria encoded genes in the PFC of mCRS mice.**

The complex I genes *mt-Nd1* and *mt-Nd3* were selected to technically verify the BRB-seq results for the mtDNA encoded genes in the PFC of mCRS and CTR mice by qRT-PCR. In addition, the complex V gene *mt-ATP6* was analyzed which was not detected via BRB-seq. *Gapdh* served as a negative control. Unpaired T-test, two-tailed; n=16/group. Data are displayed as mean ± SEM.*, $p < 0.05$.

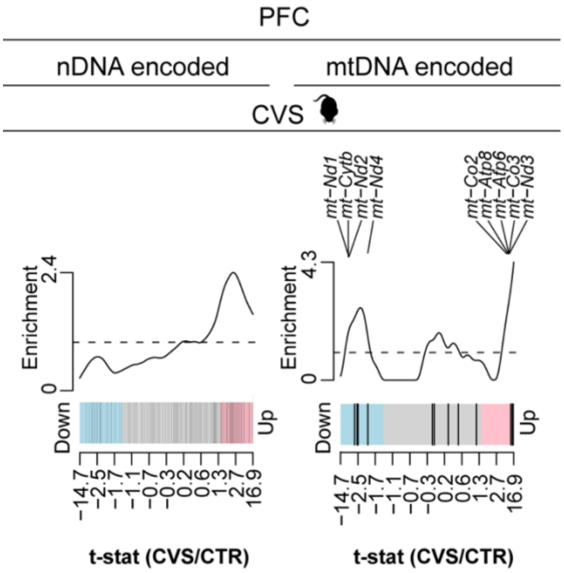


**Figure S4. Mitochondria-associated genes in the PFC of mice exposed to chronic variable stress.**

Barcode plots for mitochondria-associated genes encoded by nDNA or mtDNA DNA in the PFC of mice exposed to chronic variable stress (CVS) and corresponding unstressed CTR animals (PFC: n=19/group; NAc: n=20/group). CVS exhibit an enrichment for up-regulation of mtDNA encoded gene expression. Raw data for this analysis were obtained from a previously published data set [9].

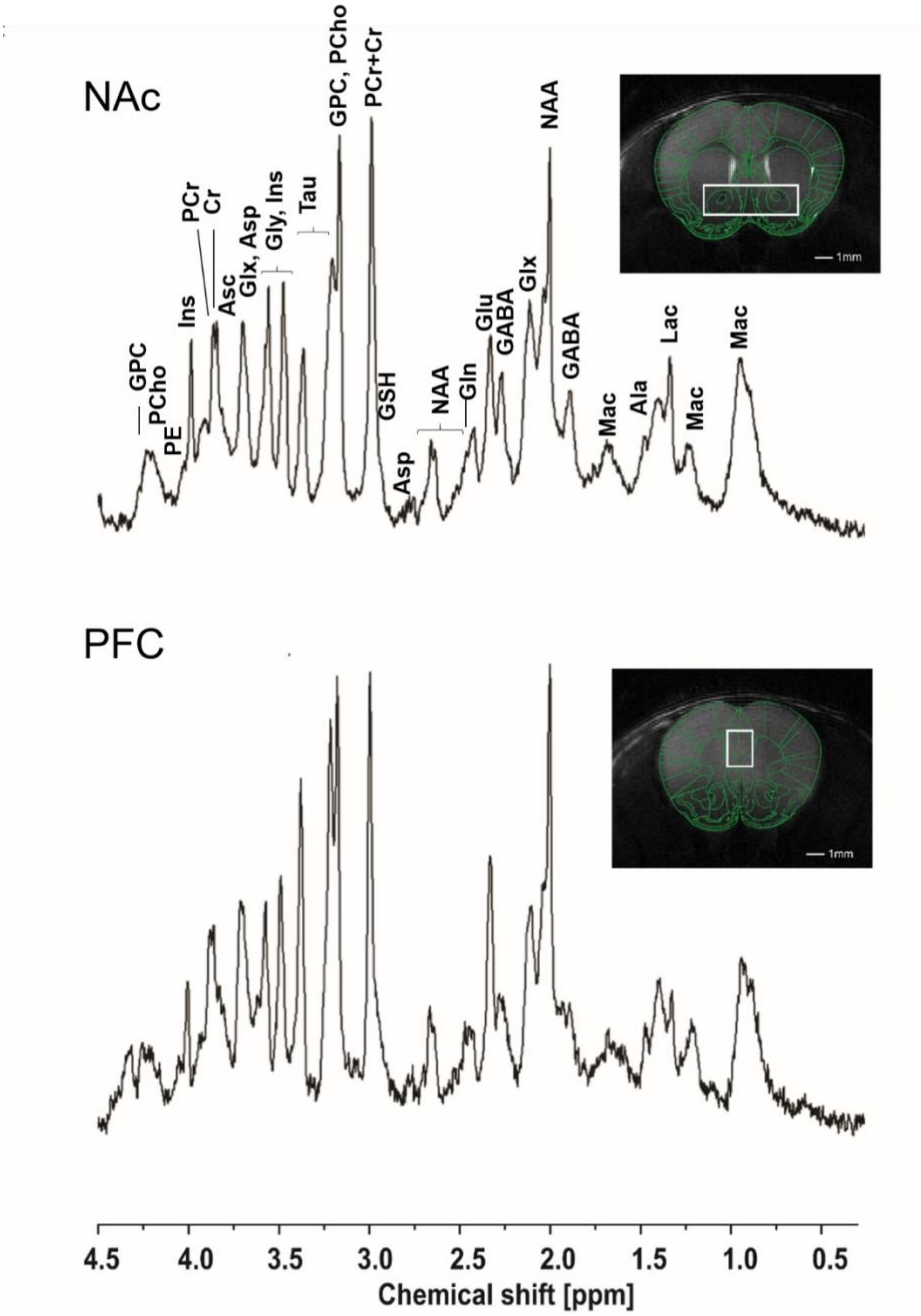


**Figure S5. Spectra of PFC and NAc in mice.**

Typical spectra of mouse NAc (top) and PFC (bottom) with location of the related volume of interest (VOI; white rectangle) on the anatomical images. The VOIs were 1.4 x 1.7 x 1.2 mm3 for PFC and 4.1 x 1.4 x 1.2 mm3 for NAc. Spectra are depicted with 2 Hz apodization. The fitted neurochemical profile included following metabolites: taurine (Tau), creatine (Cr), phosphocreatine (PCr), glutamate (Glu), γ-aminobutyric acid (GABA), aspartate (Asp), glutamine (Gln), N-acetyl-aspartate (NAA), myo-inositol (Ins), glucose (Glc), ascorbate (Asc), glutathione (GSH), phosphorylcholine (PCho), glycerophorphorylcholine (GPC), lactate (Lac), N-acetylaspartyl-glutamate (NAAG), phosphoethanolamine (PE), alanine (Ala), glycine (Gly), as well as macromolecules (Mac).

## Supplementary Tables

**Table S1. Differential expressed genes in the PFC and NAc upon mCRS.**

**Table S2. Gene enrichment analysis in the PFC and NAc upon mCRS.**

## Supplementary References


1. Ota, K. T. *et al.* REDD1 is essential for stress-induced synaptic loss and depressive behavior. *Nat. Med.* **20**, 531–5 (2014).
2. Shariq, A. S. *et al.* Evaluating the role of orexins in the pathophysiology and treatment of depression: A comprehensive review. *Prog. Neuropsychopharmacol. Biol. Psychiatry* **92**, 1–7 (2019).
3. Brundin, L., Björkqvist, M., Petersén, A. & Träskman-Bendz, L. Reduced orexin levels in the cerebrospinal fluid of suicidal patients with major depressive disorder. *Eur. Neuropsychopharmacol.* **17**, 573–9 (2007).
4. Hyde, C. L. *et al.* Identification of 15 genetic loci associated with risk of major depression in individuals of European descent. *Nat. Genet.* **48**, 1031–6 (2016).
5. Okura, T. *et al.* Mianserin suppresses R-spondin 2-induced activation of Wnt/β-catenin signaling in chondrocytes and prevents cartilage degradation in a rat model of osteoarthritis. *Sci. Rep.* **9**, 2808 (2019).
6. Okamoto, H. *et al.* Wnt2 expression and signaling is increased by different classes of antidepressant treatments. *Biol. Psychiatry* **68**, 521–7 (2010).
7. Zhou, W.-J. *et al.* The antidepressant roles of Wnt2 and Wnt3 in stress-induced depression-like behaviors. *Transl. Psychiatry* **6**, e892 (2016).
8. Matrisciano, F. *et al.* Induction of the Wnt antagonist Dickkopf-1 is involved in stress-induced hippocampal damage. *PLoS One* **6**, e16447 (2011).
9. Labonté, B. *et al.* Sex-specific transcriptional signatures in human depression. *Nat. Med.* **23**, 1102–1111 (2017).
